# Food Allergies and Quality of Life among School-Aged Children and Adolescents: A Systematic Review

**DOI:** 10.3390/children10030433

**Published:** 2023-02-23

**Authors:** Artemis-Eirini Drakouli, Ioanna Kontele, Dimitrios Poulimeneas, Stella Saripanagiotou, Maria G. Grammatikopoulou, Theodoros N. Sergentanis, Tonia Vassilakou

**Affiliations:** 1 Department of Public Health Policy, School of Public Health, University of West Attica, 196 Alexandras Avenue, 11521 Athens, Greece; 2 Department of Nutrition and Dietetics, University of the Peloponnese, 24100 Kalamata, Greece; 3 Department of Nutrition and Dietetics, Harokopio University, 17676 Athens, Greece; 4 Center of Education and Training in Eating Disorders, 14231 Athens, Greece; 5 Unit of Immunonutrition and Clinical Nutrition, Department of Rheumatology and Clinical Immunology, Faculty of Medicine, School of Health Sciences, University of Thessaly, Biopolis, 41110 Larissa, Greece

**Keywords:** health-related quality of life, quality of life, food allergy, food allergen, food challenge, immunonutrition, emotional impact, food anxiety, immunotherapy

## Abstract

Recently, besides the focus on the medical diagnosis and therapeutic interventions for food allergy (FA), the psychosocial aspects of this frequent condition have also been investigated. The current systematic review aimed to explore and synthesize the scientific evidence published from January 2015 to April 2022 on Health-Related Quality of Life (HRQoL) among children and adolescents with FAs. Twenty-eight research studies were included in the review, which was conducted on three databases (PubMed, Scopus and Cochrane Library). In most studies, the scores indicate an average level of HRQoL for children and adolescents with FAs, with girls and older children being more negatively affected than boys and younger ones, respectively. Few studies compared HRQoL between children with FA and healthy children, with 3 of them showing worse HRQoL for children with FAs. Immunotherapy has been found to improve the QoL of children with FAs. Anaphylaxis history, number of FAs, additional allergies, number and severity of symptoms were identified as the main factors with a negative impact on QoL. More comparative studies on the HRQoL of children and adolescents with FAs and healthy populations or children with other chronic diseases are required in order to improve QoL of children with FAs.

## 1. Introduction

The etymology of the word “allergy” comes from the Greek words “allos” (other, different from normal) and “ergo” (work, action of the organism). Therefore, allergy means “wrong action” [1]. Allergic reactions may range from mild local symptoms, such as Oral Allergy Syndrome (OAS), to severe life-threatening anaphylaxis [2,3]. Symptoms involve the gastrointestinal, respiratory and cardiovascular systems and the skin [2,4], while their appearance is not dose-dependent [5]. The diagnosis of suspected food allergy (FA) can be made by clinical history and physical examination, exclusion diets, Skin Prick Tests (SPT), blood test to determine specific immunoglobulin (IgE) levels and Oral Food Challenge (OFC) [5,6,7,8,9,10,11].

Epidemiological data show an increase in the prevalence of food allergy [12,13,14,15]. More common allergenic foods are peanuts and tree nuts, while allergies to milk, egg and fish are also very common [16,17]. Management of FAs is based on careful elimination of the allergenic food from the diet and prompt application of therapeutic measures to treat severe reactions in cases of accidental exposure. Immediate therapeutic interventions include epinephrine injection, antihistamines and corticosteroids [18,19,20]. However, there is no definitive treatment, and the most effective management is based on patient education [21]. New promising therapeutic approaches include Food Allergy Immunotherapy (FA-AIT) which, depending on the method of administration, is divided into Oral Immunotherapy (OIT), Sublingual Immunotherapy (SLIT) and Epicutaneous (on the skin) Immunotherapy (EPIT) [22,23,24,25]. Moreover, new trials examine the use of probiotics, modified food proteins, DNA vaccines and fecal microbiome transplantation [22,26].

Individuals with FA experience increased use of healthcare services, financial burden and significant reductions in their quality of life [17]. The Quality of Life (QoL) is a multidimensional concept and it is affected by the multiplex interactions of individual life circumstances, personal experiences and values [27]. According to World Health Organization, the individual’s QoL is defined as “the perception of their position in life in the context of the culture and value systems in which they live and in relation to their goals, expectations, standards and concerns” [28]. Health-Related Quality of Life (HRQoL) concerns the functional impact of a disease or disability and its treatment on the individual’s QoL. It is an important and meaningful outcome measure for people with FAs, and it can help in clinical decisions, including health guidelines [29].

The diagnosis of FA may affect the QoL of both the child and the caregiver and may lead to undesirable stigmatization and bullying, anxiety, depression, post-traumatic stress, financial issues and reduced QoL [30,31,32,33,34]. More time is required for food preparing, while eating out options and family activities are limited, which significantly worsens the well-being of all family members [35,36,37]. FAs may cause a lot of stress to children, especially in occasions where the caregiver is not present and the child has to decide by himself what is safe to eat [36]. Adolescents with FAs experience more school absences, reduced self-confidence and clinically worse HRQoL [38,39].

During the last decades, specific assessment tools have been developed and validated in order to assess the QoL of children with FAs which, due to the self-completion by children and adolescents or the parallel completion by parents on their behalf (proxy), provide significant information regarding the problems that children with FAs and their families face in their daily lives [40].

The aim of the present systematic review is to explore the relationship between HRQoL and FAs in school-aged children and adolescents, using general and disease-specific questionnaires. As a sub-objective, we also sought to investigate whether HRQoL scores change after therapeutic interventions, such as OFC and OIT, and to explore confounding factors that affect the HRQoL of this population.

## 2. Materials and Methods

### 2.1. Literature Search Strategy

A systematic review was performed on 30th of April 2022 in three bibliographic databases, more specifically in PubMed, Scopus and Cochrane library. This systematic review was performed according to the 2020 PRISMA guidelines [41]. The systematic review has been registered at OSF. In order to search for studies relevant to the topic of the review, the terms shown in Table 1 were combined. Moreover, the PICOS algorithm that was applied is shown in Table 2.

The reference lists of eligible papers and relevant reviews were also meticulously searched in order to include additional studies reporting on QoL among children and adolescents with FAs.

### 2.2. Eligibility Criteria

Articles eligible to be included in this review were required to meet the inclusion criteria as they are shown in Table 3, while articles meeting the exclusion criteria were excluded from the review. All article abstracts were screened by three authors (A.D., I.K. and D.P.), working in pairs in a blinded fashion. Those found not complying with the inclusion criteria were removed and any controversies were dealt with consensus in a meeting, in which the abstracts were reviewed.

### 2.3. Quality Assessment

All observational studies were rated with the Newcastle–Ottawa scale (NOS) and its versions, adapted for assessing the quality of non-randomized cross-sectional, case-control and cohort studies. This scale allocates a maximum of 10 stars, evaluating selection (representativeness, sample size, non-respondents and ascertainment of exposure), comparability and outcome (assessment, statistical test) [42]. For interventional studies, the revised Cochrane ROB2 tool [43], which assesses five parameters: random sampling, intervention methodology, missing data, outcome assessment and presentation of results, was used.

### 2.4. Data Collection Process

Data were extracted from each study in a structured coding scheme using Excel and included name of first author, year of publication, country, study design, sample size and age of children/adolescents. Moreover, the method for the FA diagnosis definition was recorded, as well as the instruments used to assess HRQoL. It was also reported whether each QoL questionnaire was completed by the children or by their parents as proxies. Additionally, potential confounding factors were noted. Finally, total QoL score and subdomain scores were extracted separately for each group (children, adolescents, parents as proxies).

### 2.5. Compliance with Ethics Guidelines

This article is based on previously conducted studies. The study is performed in accordance with the Preferred Reporting Items for Systematic Reviews and Meta-Analysis (PRISMA) guidelines [44].

## 3. Results

### 3.1. Eligible Studies

**Figure 1:**
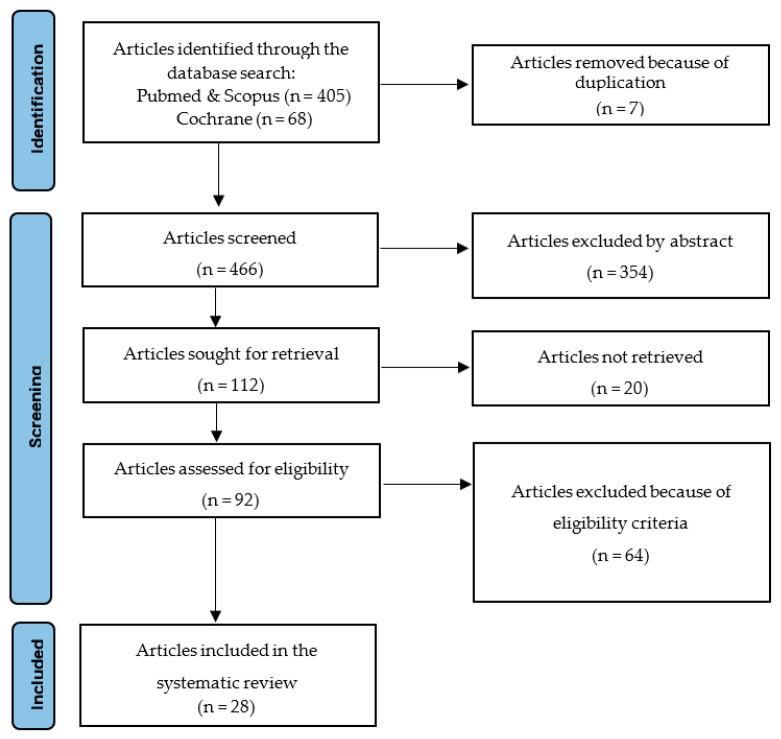
Flow diagram of the study selection process.

The initial database search retrieved 473 abstracts, of which 405 were from PubMed and Scopus, and 68 were from the Cochrane library. After removing 7 duplicated articles, we screened the remaining and 354 articles were rejected based on their abstracts, which were incompatible with the research questions of the paper. Subsequently, 92 full-text articles were retrieved of which, after careful evaluation, 64 articles were excluded based on the inclusion and exclusion criteria. Finally, 28 articles were selected for inclusion in the present work. The PRISMA 2020 flow chart, describing the sequential steps for selecting studies, is presented in Figure 1.

### 3.2. Characteristics of Eligible Studies and Population

Eighteen studies were conducted in Europe, (5 in Sweden [39,45,46,47,48], 3 in Spain [49,50,51], 3 in the Netherlands [52,53,54], 1 in Germany [55], 1 in France [56], 1 in the UK [57], 1 in Norway [58], 1 in Denmark [37] and 1 in Greece [59], while 1 study included participants from multiple European countries [60]), 5 in North America (4 in USA [61,62,63,64] and 1 in Canada [65]), 2 in Israel [66,67], 1 in Russia [68], 1 in Turkey [69] and 1 in Japan [70]. Fifteen studies were cross-sectional, 4 were case-control studies, 4 had a prospective cohort design and 5 were interventional studies (Table 4 and Table 5).

The sample size of the studies ranged between 18 and 1029 participants. In 9 of the studies, mean age of schoolchildren and/or adolescents was not mentioned separately. In the rest of the studies, mean age ranged from 6.0 to 15.9 years old. Five studies evaluated HRQoL in children, adolescents and parents as proxies at the same time [37,51,53,62,68]. Nine studies used only parents as proxies to collect data on their children’s HRQoL [39,48,49,57,63,65,66,69,70]. In 4 studies, only adolescents who answered by themselves participated [45,46,47,64] while, in 1 study, only children that answered by themselves were included [59]. Finally, in 5 studies, children and parents as proxies answered the questionnaire [50,54,55,58,67], in 2 studies, adolescents and parents as proxies were included [52,61] and, in 2 studies, the sample consisted of children and adolescents who answered by themselves [56,60].

Methods for diagnosis of FA were heterogeneous. In 11 studies, a physician’s diagnosis was used [46,50,52,54,57,59,61,63,64,65,70]. In 7 studies, diagnosis was based on the clinical history and a positive SPT or food-specific serum/blood IgE results [39,51,53,56,58,62,68]. In 9 studies, a positive OCF was also included in the criteria for diagnosis [37,45,48,49,55,60,66,67,69]. In 1 study, a clinical examination including a structured interview, along with the high-specific IgE to the culprit food, was needed [47].

The studies used generic and disease-specific questionnaires in order to examine HRQoL of children, adolescents and parents as proxies. Generic questionnaires that were used are Pediatric Quality of Life Inventory 4.0 (PedsQL 4.0), EuroQoL-5 Dimension (EQ-5D), KIDSCREEN-52 and Child Health Questionnaire-Child Form 87 (CHQ-CF87). Seven studies used the above-mentioned generic questionnaires [47,48,56,57,58,59,64]. All the studies, except one [58], used disease-specific questionnaires, and specifically the Food Allergy Quality of Life Questionnaire (FAQLQ) in its different versions (Child Form, Teenager Form, Parent Form), according to the study’s population. Moreover, 12 studies also used the Food Allergy Independent Measure (FAIM), which examines whether the FAQLQ measures those aspects of QoL that are specifically affected by FA, rather than from other general aspects of QoL.

### 3.3. Quality of Life of Children and Adolescents with Food Allergy in Observational Studies

As shown in Table 6, seven out of the 23 observational studies indicated HRQoL scores of children and/or adolescents with FA, without comparing them with other groups [39,49,52,57,59,68,69]. Several studies found that children and adolescents with FA have QoL scores over median, and close to the European general population average [49,57,59,68]. However, other studies have reported a poor HRQoL among children and adolescents with FAs [37,45,46,63].

A difference in HRQoL between males and females has also been identified. Girls with FAs seem to have worse HRQoL compared to boys, especially with regard to the emotional impact [37,45,46,47,63]. Moreover, a significant number of studies suggests that adolescents with FAs are affected more than younger children in terms of QoL [37,61].

A total of 5 studies compared the HRQoL of children and adolescents with FAs to that of healthy controls, either by using data of previous studies and registries, or by conducting a case-control analysis [47,48,56,64,70]. In a study of parents that answered as proxies on behalf of their children with FAs and parents of children without FAs, Protudger found that cases had worse HRQoL compared to controls [48]. Accordingly, Mizuno found that HRQoL scores of children with FAs were significantly higher (indicating worse HRQoL) than the scores of children without FAs [70], although it should be noted that only parents answered the questionnaires as proxies. On the other hand, in a recent study in France, Frachette et al. [56] indicated that children and adolescents with FAs had better HRQoL than healthy controls in the domains of behavior, bodily pain, family activities and mental health, and worse only in the general health perception domain. Finally, Strinnholm [47] failed to observe any differences in the HRQoL between adolescents with or without food hypersensitivity, while median scores of adolescents with FAs were above the population norm.

A small number of studies have also compared HRQoL between patients of different types of FAs or other diseases. Soller [65] compared the HRQoL of children with peanut, sesame, and seafood allergy, but did not find statistically significant differences. On the other hand, Nowak-Wegrzyn [64] compared HRQoL scores of adolescents with peanut allergy with scores of a sample of children with chronic health conditions, such as asthma or diabetes, and found worse scores in the sample of adolescents with peanut allergy. However, the scores did not exceed the minimal important difference, indicating that adolescents with FAs may be clinically similar to adolescents with other chronic health conditions regarding HRQoL [64]. Finally, Frachette [56] suggested that children and adolescents with FAs exhibit better HRQoL compared to patients with other chronic disease, and notably diabetes.

In studies conducted in Israel, Epstein-Rigbi [66,67] examined HRQoL between children with FAs who underwent OIT and those who did not and found that HRQoL of children who underwent OIT improved significantly 6 months after OIT initiation, while there was no significant improvement in the control group of children with FAs that did not undergo OIT. It should also be noted that parents reported better QoL scores compared to their children at all stages of OIT [66,67]. Accordingly, in a Spanish study of children with egg allergy who underwent OIT, significant improvements were reported in all HRQoL domains, except emotional impact, with children expressing greater improvements than their parents as proxies [50].

One study examined HRQoL between children and adolescents who chose to undergo tree nut OFC and those who chose complete nut avoidance. When parents were examined as proxies of their children, no significant differences were observed. However, children that answered by themselves presented worse HRQoL if they had undergone the OFC compared to children with nut avoidance. Results were opposite in adolescents, with those with OFC having better HRQoL compared with their peers who avoided nuts [62].

Finally, an interesting finding comes from the recent study of de Weger [54] on children and their parents, who were recommended to introduce peanuts or tree nuts at home. It was revealed that children and parents who declined the advice had higher HRQoL scores, indicating worse QoL, compared to those who accepted those allergenic foods’ introduction [54].

### 3.4. Quality of Life of Children and Adolescents with Food Allergy in Interventional Studies

Interventional studies that have assessed the HRQoL of children and adolescents with FAs, have also compared patients that underwent OIT, as well as those who took placebo. Moreover, differences before and after the IOT or the OFC have also been examined (Table 7).

Several double-blind, placebo-controlled randomized trials have revealed significant results. Reier-Nilsen suggested that children with peanut FA demonstrate improved HRQoL two years post-OIT, while controls did not experience improvement. However, in this study, parent-proxy scores were improved to a greater extent compared with the scores of children [58]. Blumchen revealed a significant improvement in HRQoL regarding the emotional impact domain and risk of accidental exposure in children with nut allergies when compared with the placebo group [55]. Similarly, Fernandez-Rivas supported the daily administration of therapeutic maintenance doses of peanut allergen powder (PTAH) in children with peanut allergy, as continued improvements in HRQoL were observed 1.5 and 2 years after initiation [51]. Recently, the ARTEMIS study, which included children and adolescents with peanut allergy from 7 European countries, showed that children who received OIT reported greater improvements in FA-related QoL compared with the participants in the placebo arm. Nevertheless, the improvements were significant for the group of children and not for the group of adolescents [60]. On the other hand, van der Valk did not find statistically significant differences in the QoL of children and adolescents with cashew nut allergy who underwent OFC [53].

### 3.5. Confounding Factors That Affect HRQoL of Children and Adolescents with Food Allergy

A number of confounding factors that may affect HRQoL of children and adolescents with FA have been studied. Yilmaz, Thörnqvist and Mizuno found that HRQoL grew significantly worse with age [39,69,70], while Morou and Manso suggested that the HRQoL of children with FAs did not depend on age, gender and number of FAs [49,59]. However, he also indicated that patients with gastrointestinal, respiratory or multisystemic symptoms of FAs have worse HRQoL than those with milder skin symptoms [49]. Nevertheless, as the number of symptoms increases, the QoL worsens [46].

Many studies indicate that multiple FAs and simultaneous presence of non-food allergies seem to be associated with worse HRQoL [39,48,63,65]. Moreover, DunnGalvin reported that the number of foods avoided and the reactions’ severity are associated with greater QoL impairment [68], while Protudger indicated that allergy in common foods (milk, egg, cereals) is linked to worse QoL [46]. According to Acaster FA severity is closely linked to worse HRQoL [57].

History of anaphylaxis has been found as another factor that is associated with worse QoL in children and adolescents with FAs in a significant number of studies [48,63,65,70].

Finally, higher parental education level has been linked to better quality of life [65].

### 3.6. Quality Assessment of the Reviewed Studies

The quality of the reviewed studies was assessed with appropriate instruments, according to study design (Supplementary Tables S1–S3). Cross-sectional and case-control studies were rated mostly of low or moderate quality, suffering low scores in the sample selection domain. All cohort studies were rated of high quality. All interventional studies were rated as having a low risk of bias.

## 4. Discussion

The current systematic review examined the relationship between HRQoL and FAs in school-aged children and adolescents. The results regarding the level of impact of FAs to the QoL are inconclusive, as in the majority of studies the scores on the generic and disease-specific questionnaires are close to the median, indicating an average level of HRQoL.

Few studies compared the scores for QoL of children with FA to that of the corresponding healthy population. The results are also ambiguous, with 3 studies reporting worse QoL among children with FAs in comparison to healthy controls [48,64,70], one showing better HRQoL for the children with FAs [56], and one finding no difference between participants with and without FAs [47]. Moreover, the studies that compared HRQoL patients with different types of FAs and/or other diseases did not find statistically significant worse QoL of patients with FAs.

Other significant findings of the current review are that girls with FAs are affected more than boys, while older children seem to be also more affected, when compared to younger children [37,45,46,47,61,63]. This could be explained by the fact that adolescents eat more frequently than children out of home, and they possibly find more difficulties to follow a restrictive diet.

Regarding the therapeutic interventions’ impact on the QoL, the majority of studies show significant improvements after the administration of OIT, in contrast to the control or placebo groups that experience no significant changes during the same period [55,58,60].

Finally, regarding the factors that affect HRQoL alongside the FAs, history of anaphylaxis, as well as the number of allergies and the severity of symptoms, seem to be the most important ones [39,46,48,49,57,63,65]. However, social factors, such as parental education and financial status, are not thoroughly studied.

Similar findings have been reported in previous systematic reviews [40,71]. Morou indicated that HRQoL of children with FAs may differ from that of the normative population in certain subdomains, including bodily pain, physical functioning, mental health, general health, and emotional, social and psychological QoL. However, children with FAs performed better in physical health, and had fewer limitations in schoolwork due to behavioral problems [40]. Golding concluded that FA has a negative impact on children’s and adolescents’ HRQoL. Also, in agreement with the current review, it was found that adolescents have lower HRQoL than children, and that the number of allergies and the severity of FA symptoms influence HRQoL of children with FAs.

The present systematic review has a number of limitations that should be discussed. First of all, it should be noted that there are many differences regarding the FA definition and diagnosis. Several studies did not use specific serum IgE tests or food challenges to make a diagnosis and, therefore, participants in many studies may not have confirmed FA. While a food-allergen-specific analysis was intended, the original studies did not provide such sensitivity analyses; therefore, this review cannot answer whether the HRQoL of children with allergy to a specific food may be more affected. Furthermore, a wide variety of HRQoL instruments was used in the reviewed studies, all with different classification scores and ranking systems. This greatly impacts the heterogeneity of the results and, therefore, our systematic review cannot quantify the impact of food allergies on quality of life, but only provide a qualitative explanation of the relationship examined. Some studies used parents as proxies, and it can be hypothesized that parental reports of QoL on behalf of their children may not accurately reflect the child’s perception. In several studies, the sample size was small, resulting in an insufficient representation of people with FAs in the general population. Finally, regarding the quality assessment, it should be noted that all the included studies are mostly of moderate or lower quality; hence, conclusions may not be drawn safely.

Additional studies with comparisons between children with FAs and healthy populations, as well as with children suffering from other chronic diseases, are required. Furthermore, more studies that examine the impact of immunotherapy on HRQoL are considered significant. Adequate sample size, appropriate research design, and the use of validated questionnaires for the assessment of HRQoL should be ensured in future studies. Use of reliable methods to confirm FA, report of important clinical outcomes, consideration of potential confounding factors, and control for potential comorbidities, should also be taken into account.

Alongside this, the implementation of FA prevention measures in children should be prioritized. Prevention begins with exclusive breastfeeding and the appropriate time period (from 6 months) of introducing solid foods. Education regarding reading food labels and menus, with an emphasis on the presence of allergenic ingredients, should also be ensured. Educating all family members, school staff and restaurant personnel on how to manage a food allergic reaction in children and adolescents could also improve the QoL of sufferers. Finally, a patient with FAs should always be aware of items that may contain allergens (such as vaccines, drugs, cosmetics and toys), have a clear plan of action in case of an accidental exposure, and carry an epinephrine auto-injector or appropriate medication, if this is recommended by his physician.

Other practices that would help improve QoL of children with FAs are the implementation of a multidisciplinary approach to help families cope with the emotional, social, and financial burden, the function of a 24-h helpline with advice on managing anaphylaxis, and the implementation of school programs that aim to strengthen social and emotional skills of students with FAs and limit FA-related teasing from other children.

## 5. Conclusions

The current systematic review revealed that children and adolescents with FAs have an average level of QoL, similar to that of healthy individuals. The QoL of girls and older children seems to be more negatively affected by the burden of FAs than boys and younger children. Moreover, the QoL of children with severe symptoms, such as anaphylaxis, and of children with other co-existing allergies, is more negatively affected. Finally, therapeutic interventions, such as immunotherapy, contribute, not only in the improvement of children’s symptoms, but also in the improvement of their QoL.

However, considering that studies included in the current review are mostly of moderate or low quality, the findings should be interpreted with caution. In that context, future studies should be designed in a way that ensures proper diagnostic criteria, use of age-specific and population-specific validated instruments and adequate sample size. Moreover, for the improvement of the QoL of children and adolescents with FAs, more comparative studies on their QoL and on that of healthy individuals are required, in order to identify and target the aspects of the QoL specifically affected by the condition.

## Figures and Tables

**Table 1:** Keywords for the PubMed database.

Search String
(“food allergy” OR “food allergies”) AND “quality of life” AND (child OR children OR adolescent OR adolescents OR adolescence OR teen OR teenager OR teenagers)

**Table 2:** PICOS algorithm for systematic review.

	Participants	Intervention	Comparison	Outcomes	Study Design
Observational studies	Children and adolescents 6–18 years old, or/and their parents	Food Allergy diagnosis	Between food allergy patients and healthy population	Quality of Life or Health-Related Quality of Life	Cross-Sectional, Cohort and Case-Control Studies
Interventional studies	Children and adolescents 6–18 years old, or/and their parents	Oral Food Challenge or Oral Immunotherapy	Before and after the intervention or between groups that underwent or not an intervention	Quality of Life or Health-Related Quality of Life	Clinical Trials

**Table 3:** Inclusion and exclusion criteria.

Inclusion Criteria
Participants are diagnosed with FA. Participants are children (between 6 and 12 years) and adolescents (between 12 and 18 years of age), or when a mean age in between 6–18 years is reported. Studies that included parents who answered on behalf of their children using proxy-questionnaires were also included. Data about the correlation between FA and QoL or HRQoL should be provided. Studies that examined QoL of children with all types of allergies were included only if they reported separate data for children with FA. Studies that included groups of children and adolescents, or adults, were included in the review only when providing data on children and adolescents separately from the adults. Any strategy to diagnose FA and to assess QoL was deemed eligible. Regarding the instruments used to evaluate HRQoL, acceptable studies were considered those that used validated generic or disease-specific questionnaires, as well as questionnaires validated by other researchers or adapted from validated questionnaires. Prospective cohorts/cross-sectional/case-control and interventional studies (clinical trials) were included. Regarding the interventional studies, only studies that provided data on QoL scores before and after the intervention, or the mean change of scores, were included. The articles were written in English or Greek language. Studies were published between January 2015 and April 2022.
**Exclusion Criteria**
Case reports Review articles and medical hypotheses Animal studies Studies not declaring age groups Studies with younger children (less than 6 years old) or adults (more than 18 years) Studies which used questionnaires that did not assess directly the QoL Studies not published in English or Greek

**Table 4:** Characteristics of Eligible Observational Studies.

First Author	Country/Region	Study Design	Study Population and FA		Sample Size	Participant Age (Years)	FA Diagnosis
Miller [61]	USA	Cross- Sectional	Adolescents (13–17 y) and parents (as proxies) of children (0–12 y) with FA in peanuts, nuts, milk, egg, wheat, soya, sesame, fish, shellfish, fruit, vegetables or other foods		Teens: 24	ND	physician-diagnosed
					Parents: 150		
Dunn Galvin [68]	Russia	Cross- Sectional	Children (7–12 y), adolescents (13–17 y) and parents (as proxies) of children 7–12 y with FA in peanuts, milk, egg, hazelnut, almond, walnut, sesame, fish, shellfish, fruit, or other foods		Children: 44	9.9 ± 4.8	parent-and/or self-reported clinical history and SPT/specific IgE
					Teens: 48		
					Parents: 44		
Protudjer [45]	Sweden	Cross-Sectional	Adolescents (13–17 y) with FA in cow’s milk, hen’s egg, or wheat		57	ND	history of FA and positive OFC or high food-specific IgE
Dantzer [62]	USA	Cross- Sectional	Children (8–12 y), adolescents (13–18 y) and parents (as proxies) of children <8 y with FA who underwent OFC in the past 2 years, but were still avoiding ≥1 tree nut/peanut, or declined OFC and were avoiding all nuts		Children: *n* = 18 Teens: *n* = 10 Parents: *n* = 58	9.7	history of tree-nut allergy and positive SPT or high food-specific IgE
Manso [49]	Spain	Cross- Sectional	Parents (as proxies) of children 7–12 y with FA in eggs, nuts (including peanut), milk, fish/shellfish, fruits or other foods		N = 54	ND	positive OFC and positive SPT or high food-specific IgE
Dunn Galvin [63]	USA	Cross- Sectional	Parents (as proxies) of children 0–12 y with FA (specific FA’s not reported)		N = 1029	ND	physician-diagnosed
Stensgaard [37]	Denmark	Cross- Sectional	Children (8–12 y) and adolescents (13–17 y) with FA in peanuts, nuts eggs, hazelnuts or other foods, and their parents (as proxies)		Children: *n* = 73 Teens: *n* = 49 Parents: *n* = 143	Children: 10.33 ± 1.4	positive OFC and positive SPT or high food-specific IgE
						Teens: 14.94± 1.4	
Protudjer [46]	Sweden	Cross- Sectional	Adolescents (13–17 y) with FA in cow’s milk, hen’s egg and/or wheat		N = 58	ND	physician-diagnosed
Morou [59]	Greece	Cross- Sectional	Children (8–12 y) with FA in nuts, fish, egg, legumes, milk, cereal, shellfish, fruit, meat, dark chocolate, spices or food supplements		N = 110	10.0 ± 1.4	physician-diagnosed
Nowak-Wegrzyn [64]	USA	Cross- Sectional	Adolescents (13–17 y) with peanut FA		N = 102	14.6 ± 1.3	physician-diagnosed
Yilmaz [69]	Turkey	Cross- Sectional	Parents (as proxies) of children (7–12 y) with FA in cow’s milk, egg, hazelnut, walnut, peanut, legume, pistachio, wheat, sesame, meat, fish, cashew, pumpkin seeds, or banana		N = 25	9.3 (7.8–11.4)	positive SPT or high food-specific IgE and positive OFC, or a clear-cut history of anaphylaxis with food
Acaster [57]	U.K.	Cross- Sectional	Parents (as proxies) of children (4–15 y) with peanut FA		N = 100	9.82 ± 3.42	physician-diagnosed
Soller [65]	Canada	Cross- Sectional	Parents (as proxies) of children with peanut, sesame or seafood FA		N = 793	9.32 (6.91, 11.37)	physician-diagnosed
Thörnqvist [39]	Sweden	Cross- Sectional	Parents (as proxies) of children (0–12 y) with FA in hen’s egg, tree nuts, peanuts, or other foods		N = 63	ND	history of FA to ≥1 food and a positive ImmunoCAP test for allergen-specific IgE antibodies to the same food
Saleh-Langenberg [52]	The Netherlands	Cross- Sectional	Adolescents (13–17 y) with FA in tree nuts, peanuts, fruit, soy, milk, vegetables, shellfish, sesame, wheat, fish, or celery, who were prescribed an EAI and parents (as proxies)		N = 55	15.9 ± 1.29	physician-diagnosed
Mizuno [70]	Japan	Case- Control	Parents (as proxies) of children (0–12 y) with and without FA in egg, milk, peanut, wheat, or other foods		Cases: *n* = 25 Controls: *n* = 17	ND	physician-diagnosed
Strinnholm [47]	Sweden	Case- Control	Adolescents (12–13 y) with and without food hypersensitivity in milk, egg, cod, or wheat		Cases: *n* = 74 Controls: *n* = 209	ND	clinical examination including a structured interview, high specific IgE to the culprit food and a celiac screen test
Protudjer [48]	Sweden	Case- Control	Parents (as proxies) of children (0–12 y) with FA in hen’s egg, wheat, or milk, and without FA		Cases: *n* = 85 Controls: *n* = 94	6.0	physician-diagnosed and history of FA to ≥1 food (cow’s milk, hen’s egg and/or wheat) as ascertained either by a positive OFC or by high levels of food-specific IgE
Frachette [56]	France	Case- Control	Children (8–12 y) and adolescents (13–17 y) with FA (in peanuts, nuts, eggs, cow’s milk, kiwi, fish, goat’s milk, mustard, pine nuts, crustaceans, legumes, rosacea, wheat, soya or other foods), vs. healthy controls and children with other diagnoses	Cases: *n* = 135 Controls: *n* = 500		11.6 ± 2.49	history of FA, physical examinations, blood tests and SPT
Epstein-Rigbi [66]	Israel	Cohort	Parents (as proxies) of children (4–12 y) with FA (in milk, peanut, egg, sesame, or tree nuts) who undergo OΙΤ vs. controls	N = 223		OIT: 6.3 ± 2.3 Controls: 6.8 ± 2.3	positive OFC and positive SPT or high food-specific IgE
Epstein Rigbi [67]	Israel	Cohort	Children (8–12 y) with FA (in milk, peanut, egg, sesame, or tree nuts) who underwent OIT, vs. controls	N = 103		9.0 (8.0–11.0)	positive SPT and/or high specific serum IgE, and positive OFC or clinical history of allergic reaction in the past year
Vazquez-Ortiz [50]	Spain	Cohort	Children (8–12 y) with FA who underwent egg OIT	N = 18		9.1 ± 1.3	physician-diagnosed egg FA
de Weger [54]	The Netherlands	Cohort	Children (0–12 y) and parents (as proxies) of children with FA, recommended to introduce peanut/tree nut at home	Children: *n* = 19 Parents: *n* = 23		ND	physician-diagnosed

EAI, epinephrine auto-injector; FA, food allergy; IgE, immunoglobulin E; IQR, interquartile range; ND, no data; OFC, oral food challenge; OIT, oral immunotherapy; SD, standard deviation; SPT, skin prick test; means ± SD, or medians with their respective IQR.

**Table 5:** Characteristics of Interventional Studies.

First Author	Country/Region	Study Design	Study Population	Time of Assessment of HRQoL	Sample Size	Participant Age (Years) *	FA Definition
Reier-Nilsen [58]	Norway	Clinical trial	Children (5–15 y) with sensitization to peanut who underwent OIT vs. controls	at enrollment, after 1 year and after 2 years of OIT	N = 77	9.3	sensitization to peanut by a positive peanut SPT and/or high peanut-specific IgE or history of systemic reactions to peanuts
van der Valk [53]	The Netherlands	Clinical trial	Children (8–12 y), adolescents (13–17 y) and parents (as proxies) of children 2–12 y who underwent double-blind, placebo-controlled food challenges with cashew nut	before the challenge and 6 months after	Children: *n* = 33 Teens: *n* = 26 Parents: *n* = 84	9.0	history of FA and positive SPT or high food-specific IgE
Fernandez-Rivas [51]	Spain	Clinical trial	Children and adolescents (4–17 y) with FA in peanuts (and their parents as proxies), who underwent OIT, vs. a placebo group	at baseline, after 1 year and after 1.5 or 2 years	N = 142	10.0 (7.0–12.0)	clinical history of FA to peanuts, positive serum IgE to peanut, immunoCAP, and/or a positive SPT to peanut
Hourihane [60]	European	MC, DB, randomized, placebo-controlled trial	Children and adolescents (4–17 y) with FA in peanuts who underwent OIT, vs. a placebo group	before OIT and at the end of trial	N = 175	9.1 ± 3.7	clinical history, positive SPT, high food-specific IgE, and OFC
Blumchen [55]	Germany	MC, DB, randomized placebo-controlled trial	Children and adolescents (3–17 y) with peanut allergy who underwent OIT, vs. a placebo arm	4 weeks before the initial OFC and 4 weeks post-final OFC	N = 62	6.6 (4.8–9.8)	high serum peanut-specific IgE, and challenge-proven clinically relevant PA

DB, double-blind; FA, food allergy; IgE, immunoglobulin E; IQR, interquartile range; MC, multicenter; ND, no data; OIT, oral immunotherapy; OFC, oral food challenge; PA, peanut allergy; SD, standard deviation; SPT, skin prick test; * presented as means, means ± SD, or medians with their respective IQR.

**Table 6:** QoL of Children with FAs (observational studies).

First Author	Instrument	Population	Domain, Score Range [Worst, Best]	QoL Score				*p* Value	
Miller [61]	FAQLQ-PF FAQLQ-TF	Children (parent-proxy) and teens with FA		Children ^†^		Adolescents ^†^		*Children* vs. *Adolescents*	
			Emotional impact [7, 1]	3.1 (1.0–6.8)		3.8 (1.8–6.3)		0.02	
			Food anxiety [7, 1]	3.8 (1.0–7.5)		ΝA		ΝA	
			Social and dietary limitations [7, 1]	4 (1.0–7.0)		5.2 (2.3–7.0)		0.002	
			Total QoL [7, 1]	3.5 (1.1–6.9)		4.7 (1.9–6.8)		0.007	
			FAIM	3 (0.4–5.0)		2.7 (0.6–4.7)		0.78	
Dunn Galvin [68]	FAQLQ-PF FAQLQ-CF FAQLQ-TF	Children, parents (as proxies) and teens with FA							
				Parents ^‡^	Children ^‡^		Adolescents ^‡^		
			Total QoL [7, 1]	3.6 ± 1.3	3.9 ± 1.1		3.8 ± 1.7	ΝA	
			FAIM	3.7 ± 0.7	3.8 ± 0.8		3.6 ± 0.9	ΝA	
Protudjer [45]	FAQLQ-TF	Teens with FA		Boys ^M^	Girls ^M^	Total ^M^		*Boys* vs. *Girls*	
			Allergen avoidance and dietary restrictions [7, 1]						
				5.14	5.49	5.25		ΝD	
			Emotional impact, [7, 1]	4.35	5.30	4.65		<0.01	
			Risk of accidental exposure [7, 1]	4.46	4.42	4.45		ΝD	
			Total QoL [7, 1]	4.81	5.29	4.96		ΝD	
Dantzer [62]	FAQLQ-PF	Parents (as proxies) of children with nut FA who underwent OFC, or not		with OFC ^M^		without OFC ^M^			
			
			Emotional impact [7, 1]	3.25		3.38		ΝD	
			Food anxiety [7, 1]	3.71		3.81		ΝD	
			Social and dietary limitations [7, 1]	3.5		3.72		ΝD	
			Total QoL [7, 1]	3.45		3.61		ΝD	
	FAQLQ-CF	Children with nut allergy who underwent OFC, or not	Emotional impact [7, 1]	5.04		4.25		ΝD	
			Allergen avoidance [7, 1]	4.88		4.21		ΝD	
			Risk of accidental exposure [7, 1]	4.67		4.33		ΝD	
			Total QoL [7, 1]	4.83		4.30		ΝD	
	FAQLQ-TF	Adolescents with nut allergy who underwent OFC, or not	Emotional impact [7, 1]	3.48		5.04		ΝD	
			Allergen avoidance [7, 1]	3.86		3.97		ΝD	
			Risk of accidental exposure [7, 1]	3.86		4.05		ΝD	
			Total QoL [7, 1]	3.74		4.44		ΝD	
Manso [49]	FAQLQ-PF	Parents of children with FA							
			Emotional impact [7, 1]	2.9 ± 1.0 ^‡^					
			Food anxiety [7, 1]	3.4 ± 1.5 ^‡^					
			Social and dietary limitations [7, 1]	2.6 ± 1.2 ^‡^					
			Total QoL [7, 1]	3.0 ± 1.1 ^‡^					
Dunn Galvin [63]	FAQLQ-PF			Boys ^M^	Girls ^M^	USA ^M^	Europe ^M^		
		Parents (as proxies) of children with FA							
			Emotional impact [7, 1]	3.91	4.25	4.06	ΝD	ΝD	
			Food anxiety [7, 1]	4.27	4.63	4.42	ΝD	ΝD	
			Social and dietary limitations [7, 1]	4.29	4.52	4.39	ΝD	ΝD	
			Total QoL [7, 1]	4.16	4.45	4.29	3.8	ΝD	
Stensgaard [37]	FAQLQ-CF			Boys ^‡^		Girls ^‡^			
		Children with peanut, hazelnut or egg FA	Allergen avoidance [7, 1]	3.56±1.50		3.84±1.58		ΝD	
			Dietary restrictions [7, 1]	3.49±1.53		4.15±1.54		ΝD	
			Emotional impact [7, 1]	4.10 ± 1.68		4.52 ± 1.60		ΝD	
			Risk of accidental exposure [7, 1]	3.38 ± 1.55		3.99 ± 1.76		ΝD	
			Total QoL [7, 1]	3.64 ± 1.39		4.12 ± 1.51		ΝD	
			FAIM	3.08 ± 1.16		3.82 ± 1.39		ΝD	
	FAQLQ-TF	Adolescents peanut, hazelnut or egg FA	Allergen avoidance and dietary restrictions [7, 1]	3.66 ± 1.62		4.39 ± 1.21		ΝD	
			Emotional impact [7, 1]	3.90 ± 1.47		4.46 ± 1.21		ΝD	
			Risk of accidental exposure [7, 1]	3.59 ± 1.69		4.03 ± 1.66		ΝD	
			Total QoL [7, 1]	3.71 ± 1.51		4.32 ± 1.20		ΝD	
			FAIM	3.42 ± 1.06		3.45 ± 1.22		ΝD	
	FAQLQ-PF	Parents (as proxies) of children with peanut, hazelnut or egg FA		Fathers ^‡^		Mothers ^‡^			
			Emotional impact [7, 1]	2.76 ± 1.06		2.85 ± 1.21		ΝD	
			Food anxiety [7, 1]	3.24 ± 1.30		3.26 ± 1.38		ΝD	
			Social and dietary limitations [7, 1]	2.65 ± 1.30		2.57 ± 1.31		ΝD	
			Total QoL [7, 1]	2.89 ± 1.14		2.89 ± 1.20		ΝD	
			FAIM	3.89 ± 0.84		4.01 ± 0.89		ΝD	
	FAQLQ-PF	Parents (as proxies) of adolescents with peanut, hazelnut or egg FA	Emotional impact [7, 1]	3.04 ± 1.62		3.18 ± 1.25		ΝD	
			Food anxiety [7, 1]	3.30 ± 1.31		3.61 ± 1.41		ΝD	
			Social and dietary limitations [7, 1]	2.43±1.13		2.97±1.49		ΝD	
			Total QoL [7, 1]	2.92±1.13		3.25±1.32		ΝD	
			FAIM	3.97±0.58		4.00±0.89		ΝD	
Protudjer [46]	FAQLQ-TF			Boys ^‡^		Girls ^‡^	Total ^M^	*Boys* vs. *girls*	
		Adolescents with staple FA	Allergen avoidance and dietary restrictions [7, 1]						
				ND		ND	4.95	ND	
			Risk of accidental exposure [7, 1]	ND		ND	4.19	ND	
			Emotional impact [7, 1]	4.50±0.24		5.38±1.4	ΝD	0.04	
			Total QoL [7, 1]	4.51±1.23		5.12±1.01	4.70	0.07	
Morou [59]	FAQLQ-CF			Total ^‡^					
		Children with FA	Emotional impact [7, 1]	3.98 ± 1.21					
			Allergen avoidance [7, 1]	2.45 ± 1.26					
			Risk of accidental exposure [7, 1]	2.69 ± 1.27					
			Dietary restrictions [7, 1]	2.55 ± 1.30					
			Total QoL [7, 1]	2.92 ± 1.08					
			FAIM	2.95 ± 1.06					
	PedsQL 4.0		Physical functioning [100, 0]	91.42 ± 10.99					
			Emotional functioning [100, 0]	81.68 ± 17.86					
			Social functioning [100, 0]	87.31 ± 16.76					
			School functioning [100, 0]	89.59 ± 13.05					
			Total QoL [100, 0]	88.01 ± 11.22					
Nowak-Wegrzyn [64]	FAQLQ-TF			Total ^‡^					
		Adolescents with peanut FA	Emotional impact [7, 1]	4.9 ± 1.3					
			Allergen avoidance [7, 1]	5.0 ± 1.3					
			Risk of accidental exposure [7, 1]	5.0 ± 1.3					
			Total QoL [7, 1]	5.0 ± 1.8					
			FAIM	4.3 ± 1.2					
	PedsQL 4.0						*vs. Healthy*	*vs. other diagnoses*	
		Adolescents with peanut FA	Physical functioning [0, 100]	75.4 ± 29.4			<0.001	0.180	
			Psychosocial health [0, 100]	66.2 ± 23.4			<0.001	0.035	
			Emotional functioning [0, 100]	61.3 ± 26.7			<0.001	0.004	
			Social functioning [0, 100]	69.6 ± 27.7			<0.001	0.021	
			School functioning [0, 100]	69.6 ± 27.7			<0.001	0.804	
			Total QoL [0, 100]	69.4 ± 23.0			<0.001	0.045	
Yilmaz [69]	FAQLQ-PF	Parents(as proxies) of children with FAs		Total ^ƒ^					
			Emotional impact [7, 1]	3.1 (0.3)					
			Food anxiety [7, 1]	3.9 (0.3)					
			Social and dietary limitations [7, 1]	2.9 (0.3)					
			Total QoL [7, 1]	3.3 (0.3)					
Acaster [57]	FAQLQ-PF	Parents (as proxies) of children with peanut FA							
			Emotional impact [7, 1]	3.14 ± 1.60					
			Food anxiety [7, 1]	3.72 ± 1.65					
			Social and dietary limitations [7, 1]	3.40 ± 1.63					
			Total QoL [7, 1]	3.37 ± 1.57					
			FAIM	3.78 ± 0.89					
	EQ-5D		Total QoL [1, 0]	0.873 ± 0.231					
Soller [65]	FAQLQ-PF10	Parents (as proxies) of children with peanut, sesame and seafood FA							
			Total QoL of all patients	2.50 ± 1.37					
			Total QoL of peanut FA patients	2.53 ± 1.34					
			Total QoL of sesame FA patients	2.56 ± 1.53					
			Total QoL of seafood FA patients	1.97 ± 1.63					
Thörnqvist [39]	FAQLQ-PF	Parents (as proxies) of children with FAs							
			Emotional impact [7, 1]	2.56 ± 1.35					
			Food anxiety [7, 1]	2.48 ± 1.38					
			Social and dietary limitations [7, 1]	2.89 ± 1.56					
			Total QoL [7, 1]	2.65 ± 1.32					
Saleh-Langenberg [52]	FAQLQ-TF	Adolescents with FAs who had been prescribed an EAI	Allergen avoidance [7, 1]	4.02 ± 1.44					
			Risk of accidental exposure [7, 1]	3.92 ± 1.46					
			Emotional impact [7, 1]	3.99 ± 1.51					
			Total QoL [7, 1]	4.03 ± 1.35					
			FAIM	3.57 ± 0.96					
	FAQLQ-PF	Parents of adolescents who had been prescribed an EAI	Emotional impact [7, 1]	2.82 ± 1.02		NA		NA	
			Food anxiety [7, 1]	3.83 ± 1.08		NA		NA	
			Social restrictions [7, 1]	2.82 ± 1.02		NA		NA	
			Dietary restrictions [7, 1]	3.85 ± 1.32		NA		NA	
			Total QoL [7, 1]	3.42 ± 0.97		NA		NA	
Mizuno [70]	FAQLQ-PF	Parents (as proxies) of children with FA and controls		with FA ^‡^		Controls ^‡^		*With FAs* vs. *no FAs*	
	
			Emotional impact [7, 1]	3.6 ± 1.4		0.4 ± 0.9		<0.001	
			Food anxiety [7, 1]	4.3 ± 1.6		0.4 ± 0.9		<0.001	
			Social and dietary limitations [7, 1]	4.0 ± 1.5		0.4 ± 0.9		<0.001	
			Total QoL [7, 1]	3.8 ± 1.3		0.4 ± 0.8		<0.001	
Strinnholm [47]	FAQLQ-ΤF		Allergen avoidance and dietary restrictions [7, 1]	Boys ^M^		Girls ^M^	Total ^M^	*Boys* vs. *girls*	
				
		Adolescents with FH		3.57		3.75	3.67	0.579	
			Emotional impact [7, 1]	2.78		2.90	2.86	0.711	
			Risk of accidental exposure [7, 1]	3.66		3.97	3.84	0.324	
			Total QoL [7, 1]	3.40		3.60	3.51	0.496	
				Girls		Boys		Girls	Boys
	KIDSCREEN-52							*FH* vs. *controls*	
				with FH^Š^	Controls^Š^	with FH^Š^	Controls^Š^		
		Adolescents with FH vs. controls	Physical Well-being	49.6	49.6	49.6	49.6	0.641	0.521
			Psychological Well-being	51.8	51.7	51.8	54.5	0.447	0.172
			Moods and Emotions	50.2	54.0	54.0	55.7	0.702	0.982
			Self-Perception	49.8	52.2	52.2	55.4	0.879	0.199
			Autonomy	48.7	50.7	53.2	53.2	0.879	0.646
			Parent Relation and Home Life	54.6	54.6	54.6	54.6	0.691	0.759
			Financial Resources	56.3	56.3	56.3	56.3	0.945	0.942
			Social Support and Peers	52.4	54.9	48.3	50.2	0.667	0.828
			School Environment	54.2	54.2	52.2	52.2	0.905	0.660
			Social Acceptance and Bullying	58.8	58.8	58.8	58.8	0.037	0.947
Protudjer [48]	FAQLQ-PF	Parents (as proxies) of children with FA	Emotional impact [7, 1]	~2.9 ^M^					
			Food anxiety [7, 1]	~3 ^M^					
			Social and dietary limitations [7, 1]	~3.3 ^M^					
			Total QoL [7, 1]	~3.1 ^M^					
	EQ-5D	Parents (as proxies) of children with FA vs. controls		with FA ^M^		Controls ^M^			
		
			Total QoL [1, 0]	0.84 ^M^		0.94		<0.01	
Epstein Rigbi [67]				OIT group		controls		*baseline* vs. *6 months post-OIT/controls*	
				*mean change (95% CI) post-OIT*					
	FAQLQ-CF	Children who underwent OIT vs. controls	Emotional impact [7, 1]	−1.1 (−2.5, 0.0)		−0.3 (−0.9, 0.8)		<0.001/0.44	
			Allergen avoidance [7, 1]	−1.3 (−2.1, −0.2)		0.0 (−1.5, 0.9)		<0.001/0.64	
			Dietary restrictions [7, 1]	−0.7 (−2.5, 0.5)		−0.5 (−1.6, 0.4)		0.008/0.06	
			Risk of accidental exposure [7, 1]	−0.9 (−2.9, −0.4)		0.0 (−1.2, 0.6)		<0.001/0.44	
			Total QoL [7, 1]	−1.0 (−2.3, −0.3)		−0.2 (−0.9, 0.4)		<0.001/0.13	
				pre-OIT		post-IOT		*Pre-*vs. *post-OIT*	
	FAQLQ-PF	Parents (as proxies) of children who underwent OIT before and after OIT							
			Emotional impact [7, 1]	4.2 (3.1–4.8) ^m^		2.5 (1.8–3.6) ^m^		<0.001	
			Food anxiety [7, 1]	4.4 (3.1–5.8) ^m^		2.4 (1.4–3.6) ^m^		<0.001	
			Social and dietary limitations [7, 1]	4.0 (2.1–5.0) ^m^		1.7 (1.0–3.2) ^m^		<0.001	
			Total QoL [7, 1]	4.0 (3.2–5.0) ^m^		2.2 (1.6–3.6) ^m^		<0.001	
				Children^‡^		Adolescents^‡^			
Frachette [56]	FAQLQ-CF FAQLQ-TF	Children and teens with FA							
			Allergen avoidance [7, 1]	3.40 ± 1.65		3.83 ± 1.44		NA	
			Risk of accidental exposure [7, 1]	3.59 ± 1.55		3.39 ± 1.49		NA	
			Emotional impact [7, 1]	4.74 ± 1.51		3.74 ± 1.43		NA	
			Dietary restrictions [7, 1]	3.96 ± 1.73		ND		NA	
			Total QoL [7, 1]	3.91 ± 1.44		3.69 ± 1.27		NA	
			FAIM	3.33 ± 1.14		3.32 ± 0.98		NA	
	CHQ-CF87	Children with FAs vs. controls		with FA^‡^		Controls^‡^			
		
			Behavior [0, 100]	84.49 ± 9.63		83.75 ± 12.36		ND	
			Bodily Pain [0, 100]	79.45 ± 19.8		70.17 ± 23		ND	
			Family activities [0, 100]	91.32 ± 13.26		87.89 ± 17.5		ND	
			Family cohesion [0, 100]	79.23 ± 20.83		77.65 ± 24.66		ND	
			General health perception [0, 100]	73.64 ± 15.84		75.62 ± 16.09		ND	
			Mental health [0, 100]	80.96 ± 11.42		75.18 ± 15.61		ND	
			Physical functioning [0, 100]	90.56 ± 20.05		93.18 ± 14.17		ND	
			Role/Social limitations-Behavioral [0, 100]	93.89 ± 14.84		94.74 ± 12.8		ND	
			Role/Social limitations-Emotional [0, 100]	90.96 ± 17.36		92.31 ± 15.85		ND	
			Role/Social limitations-Physical [0, 100]	93.89 ± 15.82		94.44 ± 12.61		ND	
			Self-esteem [0, 100]	84.95 ± 12.09		83.09 ± 15.3		ND	
		Adolescents with FAs vs. controls		with FA^‡^		controls^‡^			
		
			Behavior [0, 100]	85.33 ± 11.74		79.72 ± 12.94		ND	
			Bodily Pain [0, 100]	74.77 ± 26.19		67.95 ± 23.02		ND	
			Family activities [0, 100]	91.7 ± 13.06		86.62 ± 17.92		ND	
			Family cohesion [0, 100]	79.43 ± 21.76		70.84 ± 25.7		ND	
			General health perception [0, 100]	67.98 ± 16.89		73.5 ± 15.17		ND	
			Mental health [0, 100]	78.76 ± 14.52		73.08 ± 14.95		ND	
			Physical functioning [0, 100]	96.13 ± 5.36		94.43 ± 14.09		ND	
			Role/Social limitations-Behavioral [0, 100]	97.22 ± 10.2		91.99 ± 16.35		ND	
			Role/Social limitations-Emotional [0, 100]	94.7 ± 12.79		89.16 ± 12.79		ND	
			Role/Social limitations-Physical [0, 100]	97.22 ± 11.27		94.82 ± 15.22		ND	
			Self-esteem [0, 100]	77.6 ± 16.52		74.86 ± 13.81		ND	
				OIT group		Control group		*Pre-*vs. *post-OIT*	
Epstein-Rigbi [66]	FAQLQ-PF	Parents (as proxies) of children with FA before and at 6 months post-OIT vs. controls		pre-OIT ^M^	post-OIT ^M^	pre-OIT ^M^	post-OIT ^M^		
				
			Emotional impact [7, 1]	3.7	3.32	3.6	3.7	0.001	
			Food anxiety [7, 1]	3.9	3.32	3.9	3.9	<0.001	
			Social and dietary limitations [7, 1]	3.5	2.94	3.5	3.4	<0.001	
			Total QoL [7, 1]	3.7	3.19	3.7	3.8	<0.001	
Vazquez-Ortiz [50]				pre-OIT ^M^		post-OIT ^M^		*Pre-*vs. *post-OIT*	
	FAQLQ-CF	Children with FA pre-and 12 months post-OIT	Emotional impact [7, 1]	~4.2		~4.0		0.218	
			Allergen avoidance [7, 1]	~4.3		~2.9		0.011	
			Risk of accidental exposure [7, 1]	~4.1		~3.1		0.015	
			Dietary restrictions [7, 1]	~4.5		~2.2		0.002	
			Total QoL [7, 1]	~4.2		~2.9		0.014	
	FAQLQ-PF	Parents (as proxies) of children with FA pre-and 12 months post-OIT	Emotional impact [7, 1]	~2.5		~2.9		0.823	
			Food anxiety [7, 1]	~2.8		~2.5		0.414	
			Social and dietary limitations [7, 1]	~2.4		~1.3		0.019	
			Total QoL [7, 1]	~2.8		~2.3		0.164	
de Weger [54]	FAQLQ-CF	Children with FA, recommended to introduce peanut/tree-nut at home		Accepted Introduction ^m^		Declined Introduction ^m^		*Accepted* vs. *declined*	
			Emotional impact [7, 1]	3.00 (1.50–4.33)		3.67 (2.71–4.88)		0.367	
			Total QoL [7, 1]	2.70 (1.79–3.96)		3.98 (3.10–4.26)		0.161	
			FAIM	2.33 (1.83–3.17)		2.42 (2.33–3.25)		0.580	
	FAQLQ-PF	Parents (as proxies) of children recommended to introduce peanut/tree-nut at home	Food anxiety [7, 1]	2.07 (1.38–2.78)		3.00 (2.44–4.31)		0.057	
			Total QoL [7, 1]	1.92 (1.27–2.45)		2.75 (2.19–4.17)		0.062	
			FAIM	2.80 (2.20–3.35)		3.20 (2.90–4.15)		0.014	

CHQ-CF87, Child Health Questionnaire-Child Form 87; CI, confidence intervals; EAI, epinephrine auto-injector; EoE, eosinophilic esophagitis; FA, food allergy; FAIM, Food Allergy Independent Measure; FAQLQ-PF, Food Allergy Quality of Life Questionnaire-Parent Form; FAQLQ-TF, Food Allergy Quality of Life Questionnaire-Teenager Form; FAQLQ-CF, Food Allergy Quality of Life Questionnaire-Child Form; FH, food hypersensitivity; IQR, interquartile range; NA, not applicable; ND, no data; OFC, Oral Food Challenge; OIT, oral immunotherapy; PedsQL, Pediatric Quality of Life Inventory; QoL, Quality of Life; SD, standard deviation; SEM, standard error of the mean; ^M^ Mean; ^m^ median with respective IQR; ^†^ median with range; ^‡^ mean ± SD; ^ƒ^ mean (SEM); ^Š^ Median.

**Table 7:** Quality of Life for Children with Food Allergy (interventional studies).

Study	Instrument	Population	Domain, Score Range [Worst, Best]	QoL Score				*p* Value	
Reier-Nilsen [58]	PedsQL 4.0			Pre-OIT		Post-OIT		*Pre-*vs. *post-OIT*	
				*Mean (95% CI)*					
		OIT group-children	Total QoL [0, 100]	82.1 (79.1–85.2)		86.7 (83.6–89.7)		<0.0001	
		OIT group-parents	Total QoL [0, 100]	79.8 (73.6–83.3)		88.0 (85.2–90.8)		<0.0001	
		control children	Total QoL [0, 100]	83.4 (75.4–91.4)		82.2 (76.0–88.4)		0.8	
		control parents	Total QoL [0, 100]	81.7 (74.6–88.8)		82.1 (75.8–88.4)		0.9	
				Children		Teens		*Pre-*vs. *post-OFC*	
van der Valk [53]				Pre-OFC	Post-OFC	Pre-OFC	Post-OFC		
				*Mean*				*Children*	*Teens*
	FAQLQ-CF FAQLQ-TF	Children and teens with cashew nut allergy before and 6 months after OFC	Allergen avoidance [7, 1]	3.06	3.57	3.45	3.24	0.102	0.392
			Risk of accidental exposure [7, 1]	3.5	3.79	3.31	3.14	0.34	0.591
			Emotional impact, [7, 1]	3.93	3.75	3.73	3.26	0.437	0.086
			Dietary restrictions [7, 1]	3.44	3.43	NA	NA	0.97	NA
			Total QoL [7, 1]	3.32	3.49	3.5	3.22	0.491	0.286
			FAIM	2.86	3.27	3.26	2.89	0.025	0.006
				Children		Teens		*OIT* vs. *placebo*	
Hourihane [60]				OIT	placebo	OIT	placebo		
		Children and teens who underwent OIT vs. placebo arm		*mean change post-OIT*				children	teens
	FAQLQ-CF FAQLQ-TF		Emotional impact [7, 1]	−0.88	0.01	−0.20	−0.13	0.083	0.828
			Risk of accidental exposure [7, 1]	−0.69	0.51	−0.19	0.05	0.026	0.578
			Allergen avoidance and dietary restrictions [7, 1]	−0.33	0.85	0.05	−0.26	0.011	0.433
			Total QoL [7, 1]	−0.64	0.45	−0.19	−0.05	0.015	0.640
Blumchen [55]				OIT arm		placebo arm		*OIT group* vs. *placebo group*	
				*Median (IQR) change*					
	FAQLQ-CF	Children who underwent OIT vs. placebo arm	Allergen avoidance [7, 1]	−1.9 (−3.0, −0.1)		−0.1 (−0.8, 1.1)		0.08	
			Risk of accidental exposure [7, 1]	−2.0 (−3.3, −0.9)		0.0 (−1.1, 0.8)		0.02	
			Emotional impact, [7, 1]	−1.8 (−2.8, −0.9)		−0.3 (−1.0, 0.9)		0.02	
			Dietary restrictions [7, 1]	−1.2 (−2.8, 0.2)		−0.2 (−1.3, 0.7)		0.23	
			Total QoL [7, 1]	−1.0 (−2.7, −0.5)		−0.1 (−1.2, 0.7)		0.10	
	FAQLQ-PF	Parents (as proxies) of children who underwent OIT vs. placebo arm	Food anxiety [7, 1]	−0.3 (−1.2, 0.8)		−0.1 (−0.7, 0.5)		0.61	
			Emotional impact [7, 1]	−0.2 (−1.3, 0.3)		0.2 (−0.3, 0.5)		0.17	
			Social and dietary limitations [7, 1]	−0.6 (−2.0, 0.1)		−0.1 (−0.6, 0.8)		0.16	
			Total QoL [7, 1]	−0.4 (−1.2, 0.02)		−0.2 (−0.4–0.31)		0.20	

FAQLQ-PF, Food Allergy Quality of Life Questionnaire-Parent Form; FAQLQ-TF, Food Allergy Quality of Life Questionnaire-Teenager Form; FAQLQ-CF, Food Allergy Quality of Life Questionnaire-Child Form; FAIM, Food Allergy Independent Measure; PedsQL, Pediatric Quality of Life Inventory; QoL, Quality of Life; OIT, oral immunotherapy; OFC, oral food challenge; NA, not applicable; CI, confidence interval; IQR, interquartile range.

## Data Availability

Not applicable.

## Supplementary Materials

The following supporting information can be downloaded at: https://www.mdpi.com/article/10.3390/children10030433/s1, Tables S1. Quality Assessment of the reviewed cross-sectional studies, according to the Newcastle Ottawa Scale. Tables S2. Quality Assessment of the reviewed case-control and cohort studies, according to the Newcastle Ottawa Scale. Tables S3. Quality Assessment of the reviewed interventional studies, according to the Cochrane ROB2 instrument.

## References

[1] Silverstein A.M. (2000). Clemens Freiherr von Pirquet: Explaining Immune Complex Disease in 1906. Nat. Immunol..

[2] Boardman A., Knight K., Kane P., Fitzsimons R. (2019). Recognition and Management of Food Allergy in Children. Nurs. Child. Young People.

[3] Simons F.E.R., Ardusso L.R.F., Bilò M.B., Cardona V., Ebisawa M., El-Gamal Y.M., Lieberman P., Lockey R.F., Muraro A., Roberts G. (2014). International Consensus on (ICON) Anaphylaxis. World Allergy Organ. J..

[4] Ruiz Sánchez J.G., Palma Milla S., Pelegrina Cortés B., López Plaza B., Bermejo López L.M., Gómez-Candela C. (2018). A global vision of adverse reactions to foods: Food allergy and food intolerance. Nutr. Hosp..

[5] Szépfalusi Z., Spiesz K., Huttegger I. (2015). Diagnostik Und Management von Nahrungsmittelallergien Im Kindes-Und Jugendalter. Wien. Med. Wochenschr..

[6] Ebisawa M., Ito K., Fujisawa T., Ebisawa M., Ito K., Fujisawa T., Aihara Y., Ito S., Imai T., Ohshima Y. (2020). Japanese Guidelines for Food Allergy 2020. Allergol. Int..

[7] Koletzko S., Niggemann B., Arato A., Dias J.A., Heuschkel R., Husby S., Mearin M.L., Papadopoulou A., Ruemmele F.M., Staiano A. (2012). Diagnostic Approach and Management of Cow’s-Milk Protein Allergy in Infants and Children. J. Pediatr. Gastroenterol. Nutr..

[8] Makarova S.G., Namazova-Baranova L.S., Vishneva E.A., Gevorkyan A.K., Alekseeva A.A., Petrovskaya M.I. (2015). Topical Issues of Food Allergy Diagnosis in Pediatric Practice. Ann. Russ. Acad. Med. Sci..

[9] Foong R.-X., Santos A.F. (2020). Biomarkers of Diagnosis and Resolution of Food Allergy. Pediatr. Allergy Immunol..

[10] Cox A.L., Nowak-Wegrzyn A. (2018). Innovation in Food Challenge Tests for Food Allergy. Curr. Allergy Asthma Rep..

[11] Greiwe J. (2019). Oral Food Challenges in Infants and Toddlers. Immunol. Allergy Clin. N. Am..

[12] Lieberman J., Sublett J., Ali Y., Haselkorn T., Damle V., Chidambaram A., Rosen K., Mahr T. (2018). Increased incidence and prevalence of peanut allergy in children and adolescents in the united states. Ann. Allergy Asthma Immunol..

[13] McGowan E.C., Keet C.A. (2013). Prevalence of Self-Reported Food Allergy in the National Health and Nutrition Examination Survey (NHANES) 2007-2010. J. Allergy Clin. Immunol..

[14] Peters R.L., Koplin J.J., Gurrin L.C., Dharmage S.C., Wake M., Ponsonby A.-L., Tang M.L.K., Lowe A.J., Matheson M., Dwyer T. (2017). The Prevalence of Food Allergy and Other Allergic Diseases in Early Childhood in a Population-Based Study: HealthNuts Age 4-Year Follow-Up. J. Allergy Clin. Immunol..

[15] Schoemaker A.A., Sprikkelman A.B., Grimshaw K.E., Roberts G., Grabenhenrich L., Rosenfeld L., Siegert S., Dubakiene R., Rudzeviciene O., Reche M. (2015). Incidence and Natural History of Challenge-Proven Cow’s Milk Allergy in European Children—EuroPrevall Birth Cohort. Allergy.

[16] Motosue M.S., Bellolio M.F., Houten H.K.V., Shah N.D., Campbell R.L. (2018). National Trends in Emergency Department Visits and Hospitalizations for Food-Induced Anaphylaxis in US Children. Pediatr. Allergy Immunol..

[17] Warren C.M., Jiang J., Gupta R.S. (2020). Epidemiology and Burden of Food Allergy. Curr. Allergy Asthma Rep..

[18] Davis C.M., Kelso J.M. (2018). Food Allergy Management. Immunol. Allergy Clin. N. Am..

[19] Sicherer S.H., Sampson H.A. (2014). Food Allergy: Epidemiology, Pathogenesis, Diagnosis, and Treatment. J. Allergy Clin. Immunol..

[20] Mehr S., Robinson M., Tang M. (2007). Doctor? How Do I Use My EpiPen?. Pediatr. Allergy Immunol..

[21] Jones C.J., Llewellyn C.D., Frew A.J., Toit G.D., Mukhopadhyay S., Smith H. (2015). Factors Associated with Good Adherence to Self-Care Behaviours amongst Adolescents with Food Allergy. Pediatr. Allergy Immunol..

[22] Licari A., Manti S., Marseglia A., Brambilla I., Votto M., Castagnoli R., Leonardi S., Marseglia G.L. (2019). Food Allergies: Current and Future Treatments. Medicina.

[23] Nurmatov U., Dhami S., Arasi S., Pajno G.B., Fernandez-Rivas M., Muraro A., Roberts G., Akdis C., Alvaro-Lozano M., Beyer K. (2017). Allergen Immunotherapy for IgE-Mediated Food Allergy: A Systematic Review and Meta-Analysis. Allergy.

[24] Jones S.M., Burks A.W., Dupont C. (2014). State of the Art on Food Allergen Immunotherapy: Oral, Sublingual, and Epicutaneous. J. Allergy Clin. Immunol..

[25] Yee C.S.K., Rachid R. (2016). The Heterogeneity of Oral Immunotherapy Clinical Trials: Implications and Future Directions. Curr. Allergy Asthma Rep..

[26] Albuhairi S., Rachid R. (2020). Novel Therapies for Treatment of Food Allergy. Immunol. Allergy Clin. N. Am..

[27] Siegrist J., Junge A. (1989). Conceptual and Methodological Problems in Research on the Quality of Life in Clinical Medicine. Soc. Sci. Med..

[28] World Health Organization, Division of Mental Health and Prevention of Substance Abuse (1998). WHOQOL: Measuring Quality of Life.

[29] Galvin A.D., Hourihane J.O. (2016). Health-Related Quality of Life in Food Allergy. Bundesgesundheitsblatt—Gesundh.—Gesundh..

[30] Polk B.I., Dinakar C. (2017). Patient-Centered Outcomes in Food Allergy. Curr. Allergy Asthma Rep..

[31] Feng C., Kim J.-H. (2018). Beyond Avoidance: The Psychosocial Impact of Food Allergies. Clin. Rev. Allergy Immunol..

[32] Springston E.E., Smith B., Shulruff J., Pongracic J., Holl J., Gupta R.S. (2010). Variations in Quality of Life among Caregivers of Food Allergic Children. Ann. Allergy Asthma Immunol. Off. Publ. Am. Coll. Allergy Asthma Immunol..

[33] Patel N., Herbert L., Green T.D. (2017). The Emotional, Social, and Financial Burden of Food Allergies on Children and Their Families. Allergy Asthma Proc..

[34] Sharma H.P., Herbert L.J., Ebisawa M., Ballmer-Weber B.K., Vieths S., Wood R.A. (2015). Food Allergy: Psychosocial Impact and Public Policy Implications. Food Allergy: Molecular Basis and Clinical Practice.

[35] Bollinger M.E., Dahlquist L.M., Mudd K., Sonntag C., Dillinger L., McKenna K. (2006). The Impact of Food Allergy on the Daily Activities of Children and Their Families. Ann. Allergy Asthma Immunol. Off. Publ. Am. Coll. Allergy Asthma Immunol..

[36] Walkner M., Warren C., Gupta R.S. (2015). Quality of Life in Food Allergy Patients and Their Families. Pediatr. Clin. N. Am..

[37] Stensgaard A., Bindslev-Jensen C., Nielsen D., Munch M., DunnGalvin A. (2016). Quality of Life in Childhood, Adolescence and Adult Food Allergy: Patient and Parent Perspectives. Clin. Exp. Allergy.

[38] Middelveld R., Gunnarsson N.V., Ahlstedt S., Protudjer J.L.P. (2020). Associations between Food Allergy and Perceived Life Status. Ann. Allergy Asthma Immunol..

[39] Thörnqvist V., Middelveld R., Wai H.M., Ballardini N., Nilsson E., Strömquist J., Ahlstedt S., Nilsson L.J., Protudjer J.L.P. (2019). Health-Related Quality of Life Worsens by School Age amongst Children with Food Allergy. Clin. Transl. Allergy.

[40] Morou Z., Tatsioni A., Dimoliatis I.D.K., Papadopoulos N.G. (2014). Health-Related Quality of Life in Children with Food Allergy and Their Parents: A Systematic Review of the Literature. J. Investig. Allergol. Clin. Immunol..

[41] Page M.J., McKenzie J.E., Bossuyt P.M., Boutron I., Hoffmann T.C., Mulrow C.D., Shamseer L., Tetzlaff J.M., Akl E.A., Brennan S.E. (2021). The PRISMA 2020 Statement: An Updated Guideline for Reporting Systematic Reviews. BMJ.

[42] Lo C.K.-L., Mertz D., Loeb M. (2014). Newcastle-Ottawa Scale: Comparing Reviewers’ to Authors’ Assessments. BMC Med. Res. Methodol..

[43] Sterne J.A.C., Savović J., Page M.J., Elbers R.G., Blencowe N.S., Boutron I., Cates C.J., Cheng H.-Y., Corbett M.S., Eldridge S.M. (2019). RoB 2: A Revised Tool for Assessing Risk of Bias in Randomised Trials. BMJ.

[44] Moher D., Liberati A., Tetzlaff J., Altman D.G., The PRISMA Group (2009). Preferred Reporting Items for Systematic Reviews and Meta-Analyses: The PRISMA Statement. BMJ.

[45] Protudjer J.L.P., Middelveld R., Dahlén S.E., Ahlstedt S., FoodHE Investigators (2019). Food Allergy-Related Concerns during the Transition to Self-Management. Allergy Asthma Clin. Immunol..

[46] Protudjer J.L.P., Jansson S.-A., Middelveld R., Östblom E., Dahlén S.-E., Arnlind M.H., Bengtsson U., Kallström-Bengtsson I., Marklund B., Rentzos G. (2016). Impaired Health-Related Quality of Life in Adolescents with Allergy to Staple Foods. Clin. Transl. Allergy.

[47] Strinnholm Å., Hedman L., Winberg A., Jansson S.-A., Lindh V., Rönmark E. (2017). Health Related Quality of Life among Schoolchildren Aged 12–13 Years in Relation to Food Hypersensitivity Phenotypes: A Population-Based Study. Clin. Transl. Allergy.

[48] Protudjer J.L.P., Jansson S.-A., Östblom E., Arnlind M.H., Bengtsson U., Dahlén S.-E., Kallström-Bengtsson I., Marklund B., Middelveld R.J.M., Rentzos G. (2015). Health-Related Quality of Life in Children with Objectively Diagnosed Staple Food Allergy Assessed with a Disease-Specific Questionnaire. Acta Paediatr..

[49] Manso L., Pineda R., Huertas B., Fernández-Rivas M., Diéguez M.C., Cerecedo I., Muriel A., Fernández F.B., DunnGalvin A., Antolín-Amérigo D. (2017). Validation of the Spanish Version of the Food Allergy Quality of Life Questionnaire-Parent Form (S-FAQLQ-PF). J. Investig. Allergol. Clin. Immunol..

[50] Vazquez-Ortiz M., Alvaro M., Piquer M., Dominguez O., Giner M.T., Lozano J., Jiménez-Feijoo R., Plaza A.M. (2015). Impact of Oral Immunotherapy on Quality of Life in Egg-Allergic Children. Pediatr. Allergy Immunol..

[51] Fernandez-Rivas M., Vereda A., Vickery B.P., Sharma V., Nilsson C., Muraro A., Hourihane J.O., DunnGalvin A., du Toit G., Blumchen K. (2022). Open-Label Follow-on Study Evaluating the Efficacy, Safety, and Quality of Life with Extended Daily Oral Immunotherapy in Children with Peanut Allergy. Allergy.

[52] Saleh-Langenberg J., Flokstra-de Blok B.M.J., Goossens N.J., Kemna J.C., van der Velde J.L., Dubois A.E.J. (2016). The Compliance and Burden of Treatment with the Epinephrine Auto-Injector in Food-Allergic Adolescents. Pediatr. Allergy Immunol..

[53] van der Valk J.P.M., Gerth van Wijk R., Flokstra-de Blok B.M.J., van der Velde J.L., de Groot H., Wichers H.J., Dubois A.E.J., de Jong N.W. (2016). No Difference in Health-Related Quality of Life, after a Food Challenge with Cashew Nut in Children Participating in a Clinical Trial. Pediatr. Allergy Immunol..

[54] de Weger W.W., Kunst M., Herpertz C.E.M., van der Meulen G., van Lente L., Koppelman G.H., Sprikkelman A.B., Kamps A.W.A. (2022). Low Health-Related Quality of Life Is Associated with Declining Home Introduction of Suspected Food Allergens. Clin. Exp. Allergy.

[55] Blumchen K., Trendelenburg V., Ahrens F., Gruebl A., Hamelmann E., Hansen G., Heinzmann A., Nemat K., Holzhauser T., Roeder M. (2019). Efficacy, Safety, and Quality of Life in a Multicenter, Randomized, Placebo-Controlled Trial of Low-Dose Peanut Oral Immunotherapy in Children with Peanut Allergy. J. Allergy Clin. Immunol. Pract..

[56] Frachette C., Fina A., Fontas E., Donzeau D., Hoflack M., Gastaud F., Baechler E., Dor E., Descos B., Triolo V. (2022). Health-Related Quality of Life of Food-Allergic Children Compared with Healthy Controls and Other Diseases. Pediatr. Allergy Immunol..

[57] Acaster S., Gallop K., de Vries J., Ryan R., Vereda A., Knibb R.C. (2020). Peanut Allergy Impact on Productivity and Quality of Life (PAPRIQUA): Caregiver-Reported Psychosocial Impact of Peanut Allergy on Children. Clin. Exp. Allergy.

[58] Reier-Nilsen T., Carlsen K.C.L., Michelsen M.M., Drottning S., Carlsen K.-H., Zhang C., Borres M.P., Håland G. (2019). Parent and Child Perception of Quality of Life in a Randomized Controlled Peanut Oral Immunotherapy Trial. Pediatr. Allergy Immunol..

[59] Morou Z., Vassilopoulou E., Galanis P., Tatsioni A., Papadopoulos N.G., Dimoliatis I.D.K. (2021). Investigation of Quality of Life Determinants in Children with Food Allergies. Int. Arch. Allergy Immunol..

[60] O’B Hourihane J., Beyer K., Abbas A., Fernández-Rivas M., Turner P.J., Blumchen K., Nilsson C., Ibáñez M.D., Deschildre A., Muraro A. (2020). Efficacy and Safety of Oral Immunotherapy with AR101 in European Children with a Peanut Allergy (ARTEMIS): A Multicentre, Double-Blind, Randomised, Placebo-Controlled Phase 3 Trial. Lancet Child Adolesc. Health.

[61] Miller J., Blackman A.C., Wang H.T., Anvari S., Joseph M., Davis C.M., Staggers K.A., Anagnostou A. (2020). Quality of Life in Food Allergic Children: Results from 174 Quality-of-Life Patient Questionnaires. Ann. Allergy Asthma Immunol..

[62] Dantzer J.A., Wood R.A. (2019). The Impact of Tree Nut Oral Food Challenges on Quality of Life and Acute Reactions in Nut Allergic Patients. J. Allergy Clin. Immunol. Pract..

[63] DunnGalvin A., Koman E., Raver E., Frome H., Adams M., Keena A., Hourihane J.O., Gallagher P.L., Blok B.F., Dubois A. (2017). An Examination of the Food Allergy Quality of Life Questionnaire Performance in a Countrywide American Sample of Children: Cross-Cultural Differences in Age and Impact in the United States and Europe. J. Allergy Clin. Immunol. Pract..

[64] Nowak-Wegrzyn A., Hass S.L., Donelson S.M., Robison D., Cameron A., Etschmaier M., Duhig A., McCann W.A. (2021). The Peanut Allergy Burden Study: Impact on the Quality of Life of Patients and Caregivers. World Allergy Organ. J..

[65] Soller L., Clarke A.E., Lyttle A., Chin R., Ben-Shoshan M., Cheuk S., Asai Y., Chan E.S. (2020). Comparing Quality of Life in Canadian Children with Peanut, Sesame, and Seafood Allergy. J. Allergy Clin. Immunol. Pract..

[66] Epstein-Rigbi N., Goldberg M.R., Levy M.B., Nachshon L., Elizur A. (2019). Quality of Life of Food-Allergic Patients Before, During, and After Oral Immunotherapy. J. Allergy Clin. Immunol. Pract..

[67] Epstein Rigbi N., Schwartz N., Goldberg M.R., Levy M.B., Nachshon L., Elizur A. (2021). Medical Clown Support Is Associated with Better Quality of Life of Children with Food Allergy Starting Oral Immunotherapy. Pediatr. Allergy Immunol..

[68] DunnGalvin A., Treneva M., Pampura A., Grebenko A., Makatsori M., Munblit D. (2019). Quality of Life Associated with Maternal Anxiety Disorder in Russian Children and Adolescents with Food Allergy. Pediatr. Allergy Immunol..

[69] Arik Yilmaz E., Cavkaytar O., Buyuktiryaki B., Soyer O., Sahiner U.M., Sekerel B.E., DunnGalvin A., Karabulut E., Sackesen C. (2018). Factors Affecting Food Allergy-Related Quality of Life From Parents’ Perception in Turkish Children. Allergy Asthma Immunol. Res..

[70] Mizuno Y., Ohya Y., Nagao M., DunnGalvin A., Fujisawa T. (2017). Validation and Reliability of the Japanese Version of the Food Allergy Quality of Life Questionnaire–Parent Form. Allergol. Int..

[71] Golding M.A., Batac A.L.R., Gunnarsson N.V., Ahlstedt S., Middelveld R., Protudjer J.L.P. (2022). The Burden of Food Allergy on Children and Teens: A Systematic Review. Pediatr. Allergy Immunol..

