# Supplementary material for: Food Allergies and Quality of Life among School-Aged Children and Adolescents: A Systematic Review
Source: Children (Basel). 2023 Feb 23;10(3):433. doi: 10.3390/children10030433 (PMC10047601; doi:10.3390/children10030433)
Supplement: Supplementary file 1 [file children-10-00433-s001.zip › children-2168343-supplementary.pdf]

Supplementary Table S1. Quality Assessment of the reviewed cross-sectional studies, according to the Newcastle Ottawa Scale

Study	Selection				Comparability		Outcome		Score
Miller, 2020	-	-	-	*	*	-	*	*	4
Dunn Galvin, 2020	-	-	-	**	-	-	*	*	4
Protudjer, 2019	*	-	-	**	*	-	*	*	6
Dantzer, 2019	-	-	-	**	-	-	*	*	4
Manso, 2017	-	-	-	**	-	-	-	-	2
Dunn Galvin, 2017	*	*	-	-	*	-	*	*	5
Stensgaard, 2017	*	-	*	-	*	-	*	-	4
Protudjer, 2016	-	-	-	**	*	-	*	*	5
Morou, 2021	*	-	-	-	-	-	*	*	3
Nowak – Wegrzyn et al, 2021	-	-	*	**	*	-	*	-	5
Yilmaz et al, 2018	-	-	-	**	-	-	*	*	4
Acaster et al, 2020	-	-	-	**	*	*	*	*	6
Soller et al, 2019	*	*	-	**	*	*	*	*	8
Thörnqvist et al, 2019	-	-	-	**	*	*	*	*	6
Saleh-Langenberg et al., 2016	-	-	-	**	-	-	*	*	4

Supplementary Table S2. Quality Assessment of the reviewed case-control and cohort studies, according to the Newcastle Ottawa Scale

Study	Selection				Comparability		Outcome			Score
Case-Control Studies										
Mizuno, 2017	-	-	-	-	*	-	*	-	-	2
Strinnholm, 2017	*	*	*	-	*	-	-	*	*	6
Protudjer, 2015	*	*	-	-	-	-	-	-	-	2
Frachette et al, 2022	*	-	*	*	*	-	*	*	-	6
Cohort Studies										
Epstein-Rigbi et al, 2019	-	*	*	*	*	-	*	*	*	7
Epstein Rigbi et al, 2020	-	*	*	*	*	-	*	*	*	7
Vazquez-Ortiz et al, 2015	-	*	*	*	*	-	*	*	*	7
de Weger et al, 2022	-	*	*	*	*	-	*	*	-	6

Supplementary Table S3. Quality Assessment of the reviewed interventional studies, according to the Cochrane ROB2 instrument

Study	Randomization	Methods	Missing Data	Outcome	Reporting	Overall Risk of Bias
Reier-Nilsen, 2019	Low	Some Concerns	Low	Low	Low	Low
van der Valk, 2016	Low	Some Concerns	Low	Low	Low	Low
Fernandez-Rivas, 2022	Low	Low	Some Concerns	Low	Low	Low
Hourihane et al, 2020	Low	Low	Low	Low	Low	Low
Blumchen et al, 2019	Low	Low	Low	Low	Low	Low
